# Bidirectional role of IL-6 signal in pathogenesis of lung fibrosis

**DOI:** 10.1186/s12931-015-0261-z

**Published:** 2015-08-20

**Authors:** Takeshi Kobayashi, Kensuke Tanaka, Tetsuo Fujita, Hiroki Umezawa, Hiroyuki Amano, Kento Yoshioka, Yusuke Naito, Masahiko Hatano, Sadao Kimura, Koichiro Tatsumi, Yoshitoshi Kasuya

**Affiliations:** Department of Biochemistry and Molecular Pharmacology, Graduate School of Medicine, Chiba University, 1-8-1 Inohana, Chuo-ku, Chiba, 260-8670 Japan; Department of Respirology, Graduate School of Medicine, Chiba University, Chiba, Japan; Department of Biomedical Science, Graduate School of Medicine, Chiba University, Chiba, Japan

## Abstract

**Background:**

Various signals are known to participate in the pathogenesis of lung fibrosis. Our aim was to determine which signal is predominantly mobilized in the early inflammatory phase and thereafter modulates the development of lung fibrosis.

**Methods:**

Mice received a single dose of 3 mg/kg body weight of bleomycin (BLM) and were sacrificed at designated days post-instillation (dpi). Lung homogenates and sections from mice in the early inflammatory phase were subjected to phospho-protein array analysis and immunofluorescence studies, respectively. Bronchoalveolar lavage fluid (BALF) from mice was subjected to an enzyme-linked immunosorbent assay (EIA) for interleukin (IL)-6 and evaluation of infiltrated cell populations. The effects of endogenous and exogenous IL-6 on the BLM-induced apoptotic signal in A549 cells and type 2 pneumocytes were elucidated. In addition, the effect of IL-6-neutralizing antibody on BLM-induced lung injury was evaluated.

**Results:**

Phospho-protein array revealed that BLM induced phosphorylation of molecules downstream of the IL-6 receptor such as Stat3 and Akt in the lung at 3 dpi. At 3 dpi, immunofluorescence studies showed that signals of phospho-Stat3 and -Akt were localized in type 2 pneumocytes, and that BLM-induced IL-6-like immunoreactivity was predominantly observed in type 2 pneumocytes. Activation of caspases in BLM-treated A549 cells and type 2 pneumocytes was augmented by application of IL-6-neutralizing antibody, a PI3K inhibitor or a Stat3 inhibitor. EIA revealed that BLM-induced IL-6 in BALF was biphasic, with the first increase from 0.5 to 3 dpi followed by the second increase from 8 to 10 dpi. Blockade of the first increase of IL-6 by IL-6-neutralizing antibody enhanced apoptosis of type 2 pneumocytes and neutrophilic infiltration and markedly accelerated fibrosis in the lung. In contrast, blockade of the second increase of IL-6 by IL-6-neutralizing antibody ameliorated lung fibrosis.

**Conclusions:**

The present study demonstrated that IL-6 could play a bidirectional role in the pathogenesis of lung fibrosis. In particular, upregulation of IL-6 at the early inflammatory stage of BLM-injured lung has antifibrotic activity through regulating the cell fate of type 2 pneumocytes in an autocrine/paracrine manner.

**Electronic supplementary material:**

The online version of this article (doi:10.1186/s12931-015-0261-z) contains supplementary material, which is available to authorized users.

## Background

Idiopathic pulmonary fibrosis (IPF) is a chronic, progressive disease with an extremely poor prognosis [1]. Likewise, epidemiological studies have demonstrated that the incidence and prevalence of IPF have been increasing in most western societies in recent years [2]. Although there are many ongoing clinical trials of radical treatment for IPF, there is no effective pharmacological therapy to improve the survival of patients with IPF [3].

BLM-induced pulmonary fibrosis in mice is the most common experimental model of human IPF [4]. Genetically modified mice subjected to bleomycin (BLM) instillation provide a useful target molecule for therapeutic intervention in IPF [5–8]. In these mice, fibrosis is closely linked to an inflammatory response in the lung. On the other hand, comprehensive gene expression analysis of BLM-induced fibrotic lung has revealed that two distinct groups of genes are involved in the inflammatory and fibrotic responses [9]. A reciprocal relationship between lung inflammation and fibrosis has also been reported [10]. Furthermore, most patients who present to clinicians with subjective symptoms show a reduction of forced vital capacity (FVC), indicating that fibrosis is already present [11]. Hence, whether experimental evidence-based anti-inflammatory therapy is effective against lung fibrosis remains under debate [12]. Recently, the concept that IPF results from alveolar epithelial cell injury with scant inflammation has been generally accepted [13]. Many different molecular processes such as epithelial mesenchymal transition [14], apoptosis [15], endoplasmic reticulum stress [16], telomere shortening-associated senescence [17], and hypersecretion of MUC5B caused by a point mutation in the promoter region of the gene [18] are involved in the mechanisms of epithelial injury-based fibrosis. BLM administration can recapitulate epithelial injury-induced lung fibrosis in mice [4]. Thus, to address the complex mechanisms of the pathophysiological events in the development of lung fibrosis, BLM is a useful tool.

IL-6 is a pleiotropic cytokine and functions as a proinflammatory factor as well as a profibrotic factor in BLM-induced lung fibrosis [19]. Recently, besides TGF-β/Smad3 signaling, the signaling loop of IL-6/gp130/Stat3 has been shown to play a crucial role in the pathogenesis of lung fibrosis [20]. Furthermore, blockade of the IL-6 signal during the chronic stages of lung injury shows a beneficial effect on lung fibrosis [21, 22]. In contrast, BLM-induced IL-6 has a cytoprotective effect on alveolar epithelial cells under stress with reactive oxygen species (ROS) [23]. Likewise, the IL-6/Stat3/Akt signaling axis plays a protective role in type 2 pneumocytes by regulating surfactant homeostasis [24]. These findings suggest the possibility that IL-6 also plays a protective role in epithelial injury-based fibrosis. However, this possibility has not been shown *in vivo*.

Here, we first elucidated which intracellular signal was predominantly activated at the early inflammatory stage of BLM-injured lung by a phospho-protein array and focused on the pathophysiological role of IL-6. Then, we demonstrated *in vivo* and *in vitro* that endogenous IL-6 shows a counter-effect on BLM-induced apoptosis of type 2 pneumocytes in an autocrine/paracrine manner through activation of the Stat3/Akt signaling axis. In the bronchoalveolar space, induction of IL-6 by BLM was characterized by a biphasic response. Neutralization of IL-6 at the early fibrotic stage of BLM-induced lung injury significantly ameliorated lung fibrosis. However, it is noteworthy that neutralization of IL-6 at the early inflammatory stage of BLM-induced lung injury accelerated the development of lung fibrosis.

## Methods

### Mice

Male C57BL/6 mice at 10 weeks of age were purchased from Clea Japan (Tokyo, Japan). Animals were housed in the Animal Resource Facility of Chiba University under pathogen-free conditions and cared for according to the animal care guidelines of Chiba University. The studies were performed according to an animal protocol approved by the Animal Welfare Committee of Chiba University.

### BLM-induced lung injury model

Mice under anesthesia with isoflurane inhalation were given a single intratracheal injection of BLM hydrochloride (3 mg/kg; Nippon Kayaku, Tokyo, Japan) dissolved in phosphate-buffered saline (PBS), using a Microsprayer® atomizer (PennCentury, Philadelphia, PA). Control mice received sham treatment with PBS.

### Phospho-protein array

At 3 days post-instillation (dpi), mice under anesthesia were intracardially perfused with ice-cold PBS to thoroughly wash out blood cells in the lungs and sacrificed. Lung lobes separated from the trachea and the main bronchi were homogenized in ice-cold lysis buffer [25] and centrifuged at 9000 × *g* for 15 min at 4 °C. The resulting supernatant (400 μg protein) was analyzed using a Pathscan Antibody Array kit (Cell Signaling Technology, Danvers, MA). Detection of phosphorylation of 39 proteins in the lung sample was performed according to the manufacturer’s protocol. Using a densitometer, each signal was normalized to the positive internal control included in the array membrane and expressed in arbitrary units.

### Immunofluorescence study

Mice were sacrificed at 3 or 8 dpi, and the lung lobes were fixed, dehydrated and frozen. Freshly cut lung sections (5 μm thickness) placed on poly-L-lysine-coated slides were pretreated with 1:10 FcR blocking agent (Miltenyi Biotech, Gladbach, Germany) for 10 min and reacted with various antibodies as follows: goat anti-prosurfactant protein (proSP)-C antibody (Santa Cruz Biotech., Santa Cruz, CA), rabbit anti-phospho-Stat3 (Cell Signaling Technology), rabbit anti-phospho-Akt antibody (Cell Signaling Technology), rabbit anti-Iba 1 antibody (WAKO, Osaka, Japan), rat anti-mouse IL-6 antibody (Biolegend, San Diego, CA), rabbit anti-S100A4 antibody (Abcam, Cambridge, UK) or mouse anti-smooth muscle α-actin/SMA (Sigma-Aldrich, St. Louis, MO). After staining with each appropriate fluorescein-conjugated second antibody, the sections were observed under a fluorescence microscope (Axio Imager A2, Zeiss, Oberkochen, Germany). Nuclei were stained with 4’6’-diamino-2-phenylindole (DAPI).

### Effect of IL-6 signal on BLM-induced apoptosis in A549 cells and primary cultured alveolar epithelial cells

A human lung adenocarcinoma epithelial cell line, A549, was purchased from European Collection of Cell Cultures (Salisbury, UK). Primary cultured alveolar epithelial cells were prepared from mice according to a method previously described with modifications [26]. The primary cultured cells were characterized by immunofluorescence study with rabbit anti-proSP-C antibody [27] and hamster anti-podoplanin/gp36 antibody (Abcam) in combination with Alexa Fluor 488-chicken anti-rabbit IgG and Alexa Fluor 594-goat anti-hamster IgG (Life Technologies, Carlsbad, CA). Then, SP-C^+^gp36^−^ cells (purity > 90 %) were used as alveolar epithelial cells for the following experiments.

After 24 h of serum deprivation in Dulbecco's Modified Eagle's Medium (DMEM) (WAKO) containing 0.2 % fatty acid-free BSA (Sigma-Aldrich), A549 cells and primary cultured alveolar epithelial cells on 24-well plates were stimulated with BLM (100 μg/ml) for 12 h. The cell lysates from A549 and primary cultured alveolar epithelial cells were subjected to western blot (WB) with anti-human cleaved caspase-8 (Asp391) antibody (Cell Signaling Technology) and anti-cleaved caspase-3 (Asp175) antibody (Cell Signaling Technology), respectively. To investigate the effects of human IL-6 (3 and 10 ng/ml, Peprotech, Rocky Hill, NJ), human IL-6-neutralizing antibody (2 μg/ml, clone MQ2-13A5, Biolegend), mouse IL-6-neutralizing antibody (2 μg/ml, clone MP5-20 F3, Biolegend), rat IgG1κ isotype control for IL-6-neutralizing antibodies (2 μg/ml, Biolegend), LY294002 (a PI3K inhibitor, 1 μM, Cell Signaling Technology) and S3I-201 (a Stat3 inhibitor, 1 μM, Sigma-Aldrich), each agent was added to the culture medium 30 min prior to BLM stimulation.

### Effect of IL-6 signal on BLM-induced cytokine expression in primary cultured alveolar epithelial cells

Primary cultured alveolar epithelial cells on 12-well plates were stimulated with BLM (100 μg/ml) for 12 h in the presence of mouse IL-6-neutralizing antibody (2 μg/ml) or isotype control. Then, supernatants were collected and centrifuged at 400 × *g* for 15 min at 4 °C. The resulting supernatants were subjected to Mouse Cytokine Antibody Array C1 (RayBiotech, Inc., Norcross, GA) and changes in expression levels of 22 inflammation-related proteins in the supernatant sample were evaluated. The array was performed according to the manufacturer’s instructions.

### Measurement of IL-6 level in bronchoalveolar lavage fluid

Mice with instillation of BLM were anesthetized and sacrificed on days 0, 0.5, 1, 2, 3, 5, 7, 8, 9, 10 and 11. Then, the lungs of each mouse were lavaged with 1 ml ice-cold PBS twice via the exposed trachea cannulated with a 20-gauge catheter. Collected bronchoalveolar lavage fluid (BALF) was centrifuged at 400 x *g* for 10 min. The resulting supernatant was subjected to IL-6 measurement using a mouse IL-6 ELISA MAX™ Standard (Biolegend), according to the manufacturer’s protocol.

### Effects of IL-6-neutralizing antibody on BLM-induced lung injury

Mouse IL-6-neutralizing antibody was intratracheally injected in BLM-instilled mice using a Microsprayer® atomizer. IL-6-neutralizing antibody was administered at 15 μg/body, and isotype control at 15 μg/body, for each injection.

For neutralization of IL-6 at the early inflammatory stage of BLM-induced lung injury, IL-6-neutralizing antibody was administered at 6, 30 and 54 h (0, 1 and 2 dpi) after instillation of BLM. Control mice received treatment with isotype control. Then, examination of apoptosis of type 2 pneumocytes, analysis of cell populations in BALF and histopathological study were performed. For examination of apoptosis, mice were anesthetized, sacrificed and intracardially perfused with PBS at 3 dpi, and the lungs were lavaged twice with 1 ml PBS via the exposed trachea cannulated with a 20-gauge catheter. Then, 1 ml PBS containing YO-PRO-1 (1:100, Life Technologies, Carlsbad, CA) and propidium iodide (0.5 μM, Life Technologies) was injected into the bronchoalveolar space through the catheter. After 15 min, the lungs were lavaged four times with 1 ml ice-cold PBS, and the bronchoalveolar space was filled with 4 % PFA. The lungs were dissected out, further fixed, frozen and sectioned. The population of apoptotic and necrotic cells was examined under a fluorescence microscope, and type 2 pneumocytes were confirmed by observing the phase-contrast of the same visual field. Then, the apoptotic or necrotic signal, corresponding to type 2 pneumocytes, was estimated. For evaluation of infiltrated cells, mice under anesthesia were sacrificed at 7 dpi, and the lungs of each mouse were lavaged with 1 ml ice-cold PBS twice via the exposed trachea cannulated with a 20-gauge catheter. After centrifugation of BALF, collected total cell count was measured using a hemocytometer. The differential cell count was determined by manually counting 200 cells/mouse after staining with Diff-Quick (Sysmex Co., Kobe, Japan). For histopathological studies, mice under anesthesia were sacrificed at 7 and 14 dpi. The lungs were perfused, dissected out, fixed, sectioned and stained with Masson’s trichrome to visualize fibrotic lesions. Semi-quantitative elucidation of lung fibrotic changes was performed according to the previously described method with a slight modification [28]. In brief, longitudinal sections along the central axis of the right lung (apical, azygous and diaphragmatic lobes) and the left lung were prepared, and one section from each lung was randomly selected (2 sections/mouse). Under 200x magnification, 10 fields in each section were randomly chosen, scored, and the average score was calculated.

For neutralization of IL-6 at the early fibrotic stage of BLM-induced lung injury, IL-6-neutralizing antibody was administered at 8, 9 and 10 days after instillation of BLM. Control mice received treatment with isotype control. Then, changes in body weight and survival rate were monitored until 14 dpi. At 14 dpi, mice were sacrificed under anesthesia, and the fibrotic changes in lung sections were visualized with Masson’s trichrome staining and scored according to the method described above.

### Statistical analysis

Data are expressed as mean ± S.E.M. Statistical analysis was conducted using Graphpad Prism Version 6 (GraphPad Software Inc., San Diego, CA). Statistical significance was determined by Student’s *t* test or analysis of variance (ANOVA) followed by the Tukey's test, and *p* values < 0.05 were considered significant.

## Results

### Activation of IL-6 signal in type 2 pneumocytes at early inflammatory stage of BLM-induced lung injury

**Fig. 1:**
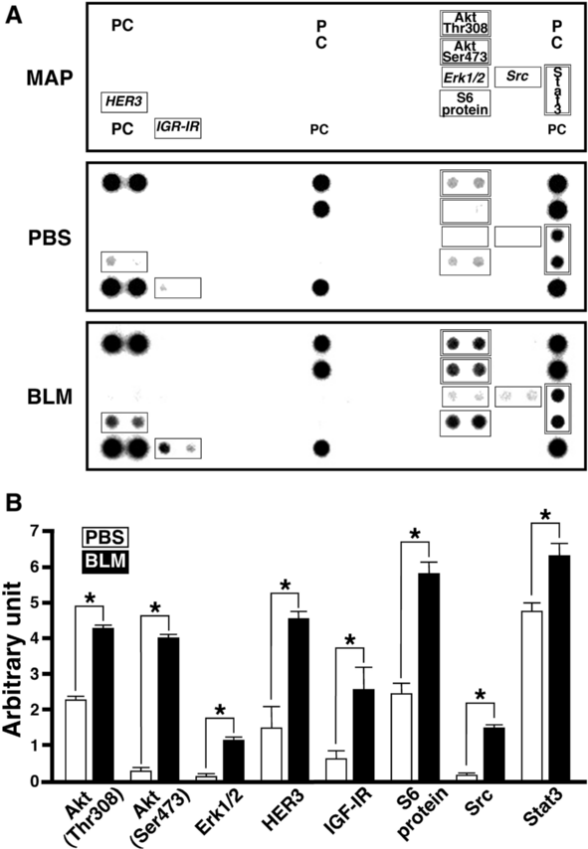
Effect of BLM on phosphorylation of signaling proteins in lung. Lysates (400 μg) from the lungs of mice instilled with PBS or BLM (3 dpi) were subjected to Pathscan Antibody Array. **a** Typical profile of BLM-induced protein phosphorylation. Proteins with increased phosphorylation levels in response to BLM are boxed. In particular, phospho-Akt and -Stat3 are boxed by a *double-line*. **b** Phosphorylation levels of these proteins in the lungs of mice instilled with PBS (white bars) and BLM (black bars). Using a densitometer, each signal was normalized to the positive internal controls included in the array glass and expressed in arbitrary units. Average signal of the positive internal controls is expressed as 10 arbitrary units. Data are shown as mean ± S.E.M. of three independent experiments. The difference between the two groups (PBS and BLM) was statistically significant (*P < 0.05) by Student’s *t*-test for unpaired values

It is well known that BLM-induced lung injury is composed of two phases, an inflammatory phase characterized by recruitment of leukocytes within one week and a fibrotic phase characterized by fibroblast proliferation and synthesis of extracellular matrix during the second week [29]. To elucidate which intracellular signal is predominantly activated at the early inflammatory stage of the BLM-injured lung, a phospho-protein array was performed. At 3 dpi, phosphorylation of several proteins such as Akt, Erk1/2, HER3, IGR-IR, S6 ribosomal protein, Src and Stat3 was induced in lung treated with BLM compared with lung treated with PBS (Fig. 1a). Phosphorylation level of each protein analyzed by a densitometer is shown in Fig. 1b. Among these proteins, we focused on Akt and Stat3 as prerequisite signaling molecules located downstream of the IL-6 receptor [30], since, in particular, little is still known about sites where IL-6 predominantly acts in early phase of BLM-induced lung injury. We examined the localization of activated forms of Akt and Stat3, and identified the cells expressing IL-6 at the early inflammatory stage of BLM-induced lung injury.

**Fig. 2:**
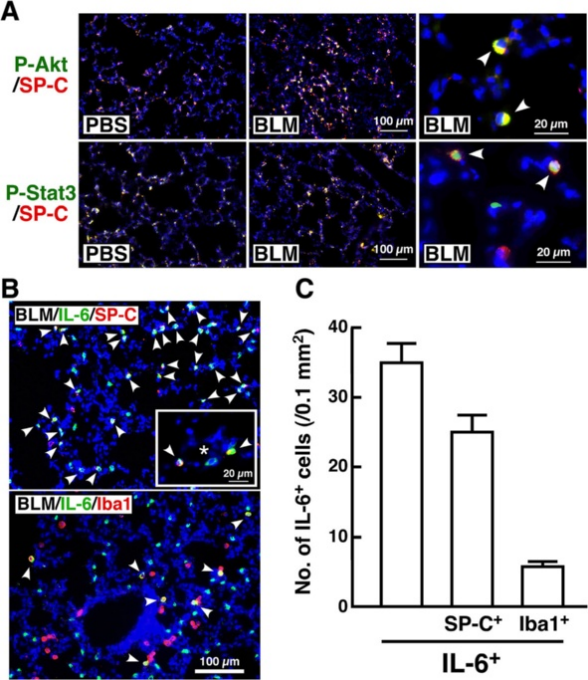
Localization of phospho-Akt and -Stat3 and expression of IL-6 at early inflammatory stage of BLM-injured lung. **a** Lung sections of mice instilled with PBS or BLM (3 dpi) were stained with anti-phospho-Akt or anti-phospho-Stat3 antibody in combination with anti-proSP-C antibody followed by reaction with Alexa 488- or Alexa 594-conjugated second antibodies (488 for phospho-Akt and -Stat3; 594 for SP-C). Arrowheads indicate phospho-Akt^+^SP-C^+^ and phospho-Stat3^+^SP-C^+^ cells. **b** Lung sections from mice instilled with BLM (3 dpi) were stained with anti-IL-6 antibody in combination with anti-proSP-C or anti-Iba1 antibody followed by reaction with Alexa 488- or Alexa 594-conjugated second antibodies (488 for IL-6; 594 for SP-C and Iba1). Arrowheads indicate IL-6^+^SP-C^+^ and IL-6^+^Iba1^+^ cells. Asterisk indicates a cell with macrophage-like morphological features. **c** Lung sections from mice instilled with BLM (3 dpi) were stained with anti-IL-6, anti-proSP-C and anti-Iba1 antibodies followed by reaction with Alexa 350-, 488- and Alexa 594-conjugated second antibodies. Data are shown as mean ± S.E.M. (*n* = 6). Nuclei were stained with DAPI. Similar results to the immunofluorescence profiles in (**a**) and (**b**) were observed in four independent experiments

As shown in Fig. 2a, phosphorylated Akt and Stat3 were mostly observed in SP-C^+^ cells in response to BLM at 3 dpi. Phospho-Akt-like immunoreactivity (LI) was detected in the cell body including the plasma membrane of SP-C^+^ cells. On the other hand, phospho-Stat3-LI overlapped with nuclei identified by DAPI. These phenomena are accordant with the facts that activated Akt is recruited to the plasma membrane, and that activated Stat3 forms a dimer and is then translocated to the nucleus [30, 31], indicating that the IL-6 signal is activated in type 2 pneumocytes at the early inflammatory stage of BLM-induced lung injury. At 3 dpi, when activation of Stat3 and Akt was observed in type 2 pneumocytes, IL-6-LI was mostly detected in SP-C^+^ cells (Fig. 2b). Besides type 2 pneumocytes, large cells showing macrophage-like morphological features in the alveolar space exhibited IL-6-LI (Fig. 2b inset). Then, whether Iba1^+^ macrophages could be positive for IL-6 was investigated. As shown in Fig. 2b, Iba1^+^IL-6^+^ cells were sparsely observed in lung sections, indicating that the main source of IL-6 could be type 2 pneumocytes but not macrophages at the early inflammatory stage of BLM-induced lung injury. By triple staining of lung sections from mice at 3 dpi with anti-IL-6, anti-proSP-C and anti-Iba1 antibodies, approximately 70 % and 14 % of IL-6^+^ cells were positive for SP-C and Iba1, respectively (Fig. 2c). We have confirmed that IL-6-LI was not observed in lung treated with PBS (data not shown). Then, we hypothesized that IL-6 induced by BLM could affect the cell fate and induce functional change of type 2 pneumocytes in an autocrine/paracrine manner and assessed this hypothesis *in vitro*.

### IL-6 has counter effect on BLM-induced cell death of type 2 pneumocytes *in vitro*

**Fig. 3:**
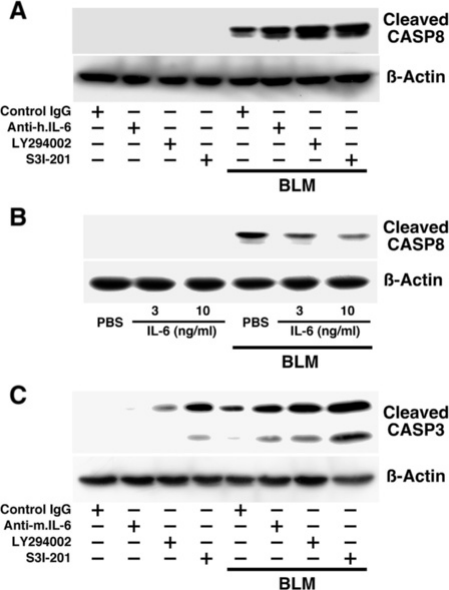
Endogenous IL-6 suppresses BLM-induced apoptotic signal in alveolar epithelial cells. **a** Effect of human IL-6-neutralizing antibody, a PI3K inhibitor LY294002, and a Stat3 inhibitor S3I-201 on BLM-induced caspase 8 activation in A549 cells. Lysates (20 μg) from cells treated with or without BLM were subjected to WB analysis for detection of cleaved caspase 8. Application of human IL-6-neutralizing antibody, control IgG, LY294002 and S3I-201 to the cells was performed 30 min prior to BLM treatment. The same lysates were subjected to WB analysis to determine the amount of β-actin as an internal control. **b** Exogenous IL-6 inhibited BLM-induced caspase 8 activation in A549 cells in a dose-dependent manner. The amount of β-actin of each lane was determined as an internal control. **c** Effect of mouse IL-6-neutralizing antibody, LY294002, and S3I-201 on BLM-induced caspase 3 activation in primary cultured SP-C^+^ cells. The basic procedure was the same as that for (**a**)

Using A549 cells, which are widely used as an *in vitro* model for type II pulmonary epithelial cells, we first investigated the effects of endogenous and exogenous IL-6 on BLM-induced activation of caspase 8, an apoptotic signal [32]. As shown in Fig. 3a, BLM increased the production of cleaved caspase 8, the activated form of caspase 8. Blockade of endogenous IL-6 by anti-IL-6 antibody augmented BLM-induced production of cleaved caspase 8. Accordingly, treatment of the cells with a PI3K inhibitor, LY294002, or a Stat3 inhibitor, S3I-201, augmented BLM-induced production of cleaved caspase 8, indicating that inhibition of the IL-6 signal induces an apoptotic signal in A549 cells. In contrast, application of IL-6 to the cells suppressed BLM-induced production of cleaved caspase 8 in a dose-dependent manner (Fig. 3b). Also in primary cultured type 2 pneumocytes, treatment of the cells with an IL-6-neutralizing antibody, LY294002 or S3I-201, augmented BLM-induced production of cleaved caspase 3, another apoptotic signal [32] (Fig. 3c). We have confirmed that application of IL-6 to both A549 cells and primary cultured type 2 pneumocytes induces phosphorylation of Akt and Stat3 in the cells (data not shown). Hence, endogenous IL-6 plays a cytoprotective role in type 2 pneumocytes through activation of Akt and Stat3 in an autocrine/paracrine manner. Then, we investigated whether endogenous IL-6 also affects cellular function of type 2 pneumocytes.

### Endogenous IL-6 modulates BLM-induced cytokine production by type 2 pneumocytes

**Fig. 4:**
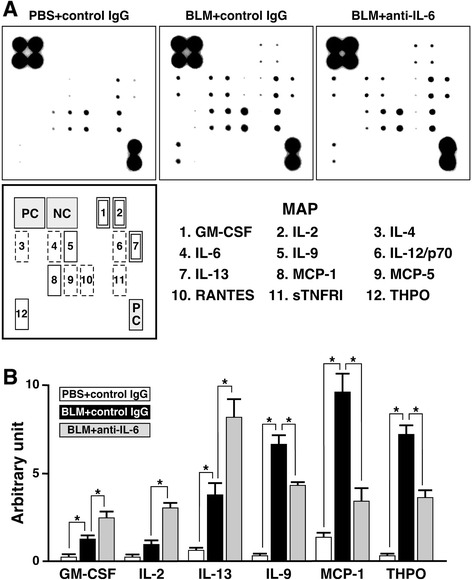
Endogenous IL-6 modulates BLM-sensitive cytokine production in type 2 pneumocytes. **a** Typical expression profile of cytokines. Primary cultured type 2 pneumocytes from mice were treated with BLM in the presence or absence of mouse IL-6-neutralizing antibody, and the supernatants were subjected to Mouse Cytokine Antibody Array C1. Application of mouse IL-6-neutralizing antibody and control IgG to the cells was performed 30 min prior to PBS or BLM treatment. The 12 molecules induced by BLM (BLM + control IgG) are numbered and indicated with the correct location in the membrane map, compared with the unstimulated control (PBS + control IgG). Among the 12 proteins, IL-6-neutralizing antibody (BLM + anti-IL-6)-induced and -reduced molecules are indicated by squares with a *double-line* and a *single-line*, respectively. IL-6-neutralizing antibody-insensitive molecules are indicated by a *broken-line*. We confirmed that the array profile of unstimulated control (PBS + control IgG) showed no difference from that of PBS alone. **b** Changes in expression of IL-6-neutralizing antibody-sensitive molecules observed in WB array analyzed by densitometer. Using a densitometer, each signal was normalized to the positive internal controls included in the array membrane and expressed in arbitrary units. Average signal of the positive internal controls is expressed as 100 arbitrary units. Data are shown as mean ± S.E.M. of three independent experiments. *P < 0.05, significantly different from value of BLM + control IgG group (ANOVA followed by Tukey's test)

It is well known that type 2 pneumocytes are involved in the intra-alveolar cytokine network [33]. Thus, we investigated whether application of IL-6-neutralizing antibody affects BLM-induced cytokine release from primary cultured type 2 pneumocytes. As shown in Fig. 4a, BLM induced the production of various cytokines/chemokines such as granulocyte macrophage colony-stimulating factor (GM-CSF), IL-2, IL-4, IL-6, IL-9, IL-12/p70, IL-13, monocyte chemoattractant protein (MCP)-1, MCP-5, regulated on activation, normal T cell expressed and secreted (RANTES), soluble tumor necrosis factor receptor (sTNFR) I and thrombopoietin (THPO). Among the BLM-induced proteins, densitometric analysis revealed that several molecules were significantly sensitive to blockade of endogenous IL-6. Blockade of endogenous IL-6 enhanced the BLM-induced production of GM-CSF, IL-2 and IL-13 but suppressed the BLM-induced production of IL-9, MCP-1 and THPO in type 2 pneumocytes (Fig. 4b).

### Time-dependent induction of IL-6 in BLM-instilled lung

**Fig. 5:**
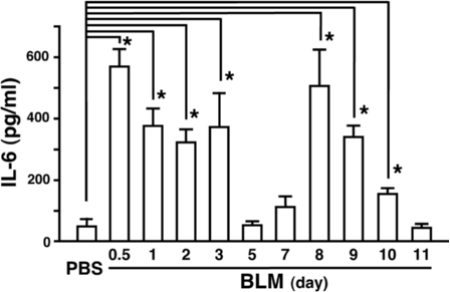
Time course of BLM-induced IL-6 level in BALF. BALF from mice treated with BLM were subjected to ELISA for mouse IL-6 at the indicated dpi. IL-6 level in BALF prepared from mice with PBS treatment is shown as negative control (PBS). Data are shown as mean ± S.E.M. (*n* = 4). *P < 0.05, significantly different from the PBS value (ANOVA followed by Tukey's test)

To elucidate how the IL-6-mediated events affect BLM-induced lung injury, the relationships between IL-6 neutralization and the pathogenesis of BLM-injured lung were evaluated. Before applying anti-IL-6 antibody to mice, the time-dependent increase of IL-6 level in BALF was observed to plan the schedule of IL-6 neutralization. As shown in Fig. 5, BLM induced a biphasic increase in IL-6 in BALF. The first peak was observed at 0.5 dpi, followed by a plateau from 1 to 3 dpi and a decline to the control level at 5 dpi; i.e., induction of IL-6 at the early inflammatory stage. The second peak was observed at 8 dpi and gradually declined to the control level at 11 dpi; i.e., induction of IL-6 at the early fibrotic stage.

### Blockade of IL-6 at early inflammatory stage of BLM-induced lung injury accelerates lung fibrosis

**Fig. 6:**
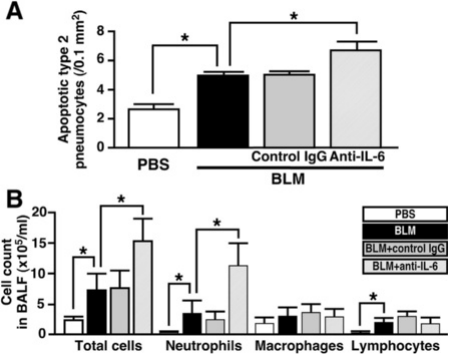
Blockade of IL-6 at early inflammatory stage of BLM-injured lung enhances apotosis of type 2 pneumocytes and alveolitis. Mice were divided into four groups: PBS group, BLM group, BLM + control IgG group, and BLM + anti-IL-6 group. **a** Apoptotic type 2 pneumocytes were elucidated by counting YO-PRO-1-permeable cells at 3 dpi. Data are shown as mean ± S.E.M. of twenty sections from each individual (n = 5). *P < 0.05, significantly different from value of BLM-treated lung (ANOVA followed by Tukey's test). **b** Effect of IL-6-neutralizing antibody on change in number of infiltrated cells into the lung. Data are shown as mean ± S.E.M. (n = 5). *P < 0.05, significantly different from value of BLM-treated lung (ANOVA followed by Tukey's test)

To block the first peak of IL-6, IL-6-neutralizing antibody was administered to mice at 6, 30 and 54 h (0, 1 and 2 dpi) after instillation of BLM. As shown in Fig. 6a, YO-PRO-1-permeable apoptotic type 2 pneumocytes increased in response to BLM at 3 dpi. BLM-induced apoptosis of the cells was significantly enhanced by treatment of the lung with IL-6-neutralizing antibody but not isotype control. On the other hand, necrotic type 2 pneumocytes stained with the intercalating agent, propidium iodide, were rarely observed, at least under our experimental conditions (data not shown). In addition, analysis of BALF revealed that infiltration of neutrophils and lymphocytes increased in response to BLM at 7 dpi. The BLM-induced neutrophilic infiltration was markedly enhanced by treatment of the lung with IL-6-neutralizing antibody but not isotype control (Fig. 6b).

**Fig. 7:**
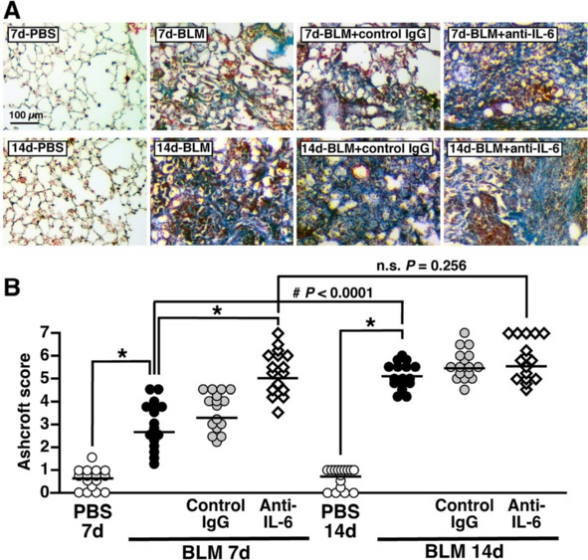
Blockade of IL-6 at early inflammatory stage of BLM-injured lung accelerates lung fibrosis. Mice were divided into four groups: PBS group, BLM group, BLM + control IgG group, and BLM + anti-IL-6 group. **a** Effect of IL-6-neutralizing antibody on BLM-induced histopathological changes in the lung. Lung sections from each group at 7 and 14 dpi were stained with Masson’s trichrome to visualize fibrotic lesions. **b** Semi-quantitative measurement of lung fibrotic change. Eight mice in each group were used. Data are shown as scores of sixteen sections (two sections/mouse). Bars represent median values. *P < 0.05, significantly different from value of BLM-treated lung (ANOVA followed by Tukey's test). The difference between the two groups (7 and 14 dpi of BLM) was statistically significant (#) by Student’s *t*-test for unpaired values. n.s., no significant difference

Histopathological findings with Masson’s trichrome staining revealed that weakly positive fibrotic lesions were sparsely observed in sections of BLM-instilled lung at 7 dpi. Treatment of BLM-instilled lung with IL-6-neutralizing antibody but not isotype control caused obvious fibrosis even at 7 dpi (Fig. 7a). This finding was supported by analysis of the modified Aschcroft scale. Compared with the PBS-instilled group, BLM modestly but significantly increased the Aschcroft score at 7 dpi, which was further increased by application of IL-6-neutralizing antibody (Fig. 7b). At 14 dpi, on the other hand, BLM induced obvious lung fibrosis, with a similar grade to that of lung treated with IL-6-neutralizing antibody or isotype control (Fig. 7a&b). In accord with those findings, the changes in SMA expression revealed that application of IL-6-neutralizing antibody markedly enhanced BLM-induced fibrogenesis especially at 7dpi (Additional file 1: Figure S1).

### Blockade of IL-6 at early fibrotic stage of BLM-induced lung injury ameliorates lung fibrosis

**Fig. 8:**
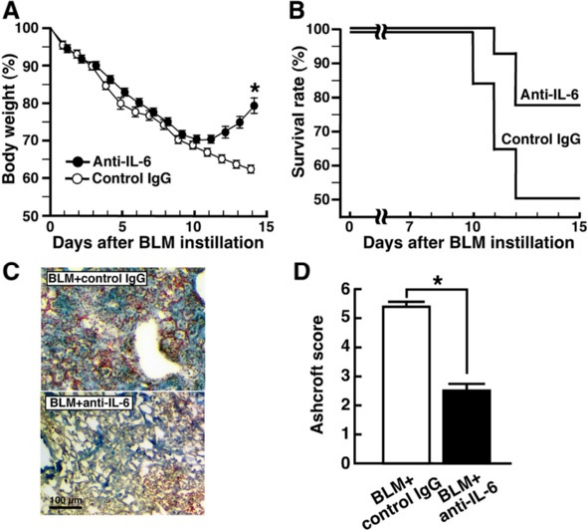
Blockade of IL-6 at early fibrotic stage of BLM-injured lung ameliorates lung fibrosis. Mice were divided into two groups: BLM + control IgG group, and BLM + anti-IL-6 group. **a** Body weight (BW) of BLM-instilled mice with or without IL-6-neutralizing antibody treatment. Time-dependent change in BW is expressed as percentage of BW of each mouse just before BLM instillation. *P < 0.05, significantly different from BLM + control IgG group at each indicated time point by Student’s *t*-test. Data represent mean ± S.E.M. (*n* = 6). **b** Survival rate of BLM-instilled mice with or without IL-6-neutralizing antibody treatment. Data represent mean ± S.E.M. (*n* = 12). **c** Effect of IL-6-neutralizing antibody on BLM-induced histopathological change in the lung. Lung sections from each group at 14 dpi were stained with Masson’s trichrome to visualize fibrotic lesions. **d** Semi-quantitative measurement of lung fibrotic change. Six mice in each group were used. Data are shown as mean ± S.E.M. of twelve sections (two sections/mouse). *P < 0.05, significantly different from BLM + control IgG group by Student’s *t*-test

**Fig. 9:**
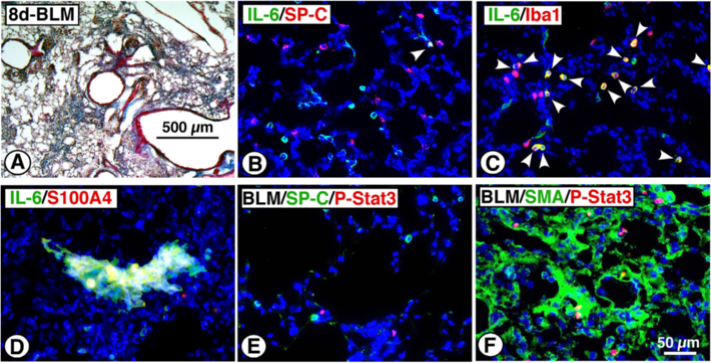
Localization of phospho-Stat3 and expression of IL-6 at early fibrotic stage of BLM-injured lung. Lung sections from mice instilled with BLM (8 dpi) were used for the following experiments. **a** Masson’s trichrome staining. **b** Lung sections were stained with anti-IL-6 and anti-proSP-C antibodies followed by reaction with Alexa 488- or Alexa 594-conjugated second antibodies (488 for IL-6; 594 for SP-C). Arrowhead indicates IL-6^+^SP-C^+^ cell. **c** Lung sections were stained with anti-IL-6 and anti-Iba1 antibodies followed by reaction with Alexa 488- or Alexa 594-conjugated second antibodies (488 for IL-6; 594 for Iba1). Arrowheads indicate IL-6^+^Iba1^+^ cells. **d** Lung sections were stained with anti-IL-6 and anti-S100A4 antibodies followed by reaction with Alexa 488- or Alexa 594-conjugated second antibodies (488 for IL-6; 594 for S100A4). **e** Lung sections were stained with anti-proSP-C and anti-phospho-Stat3 antibodies followed by reaction with Alexa 488- or Alexa 594-conjugated second antibodies (488 for SP-C; 594 for phospho-Stat3). **f** Lung sections were stained with anti-SMA and anti-phospho-Stat3 antibodies followed by reaction with Alexa 488- or Alexa 594-conjugated second antibodies (488 for SMA; 594 for phospho-Stat3). Nuclei were stained with DAPI. Similar results to the histopathological changes and immunofluorescence profiles were observed in four independent experiments

To block the second peak of IL-6, IL-6-neutralizing antibody was administered to mice at 8, 9 and 10 dpi. As shown in Fig. 8a, BLM-induced body weight loss of mice was blocked by treatment with IL-6-neutralizing antibody. Likewise, application of IL-6-neutralizing antibody improved the survival rate (Fig. 8b). In accord with those findings, BLM-induced lung fibrosis at 14 dpi was markedly alleviated by application of IL-6-neutralizing antibody, which was supported by evaluation of the Ashcroft score (Fig. 8c&d). To elucidate IL-6-expressing cells and IL-6-acting sites at the early fibrotic stage of BLM-induced lung injury, the localization of IL-6-LI and phospho-Stat3-LI in lung sections at 8 dpi was investigated. As shown in Fig. 9b, IL-6-LI was rarely observed in SP-C^+^ type 2 pneumocytes. In contrast, Iba1^+^ cells were frequently positive for IL-6, indicating that macrophages could efficiently express IL-6 (Fig. 9c). At 8 dpi, fibrotic lesions were observed in patches in lung sections stained with Masson’s trichrome (Fig. 9a). In the fibrotic areas where DAPI^+^ nuclei were condensed, S100A4^+^ foci occasionally exhibited IL-6-LI, indicating that fibroblasts could also express IL-6 (Fig. 9d). Although SP-C^+^phospho-Stat3^+^ cells were hardly observed, phospho-Stat3-LI was mostly localized in fibrotic areas where SMA^+^ myofibroblasts were extensively observed (Fig. 9e&f).

## Discussion

Comprehensive profiling of mRNA and protein expression using microarray and WB array technology is useful for evaluating the complex mechanisms of pathophysiological events and to define a novel therapeutic target in a certain disease [5, 9, 34, 35]. In addition to these analytical options, WB array for phospho-proteins is a useful tool to determine which intracellular signal is predominantly activated under a certain condition [36]. Based on the results of phospho-protein array, we focused on Akt and Stat3, downstream signaling molecules of the IL-6 receptor, for the following reasons: 1) IL-6 induced in a variety of acute and chronic inflammatory diseases plays a major role as a trigger for acute-phase protein synthesis [37]. 2) In pulmonary inflammatory diseases, bipotential functions of IL-6 in inflammation were shown using IL-6^−/−^ mice. IL-6 can mediate persistent inflammation and subsequent fibrotic changes in the lung [19]. On the other hand, IL-6 contributes to host defense against pneumococcal pneumonia through downregulating activation of the cytokine network in the lung [38]. 3) During the fibrotic stage of BLM-induced lung injury, the IL-6/Stat3 signaling axis promotes lung fibrosis [21, 22]. However, radical oxygen species (ROS) mimicking cytotoxicity of BLM induce apoptosis of several types of cells including type II alveolar epithelial cells in organotypic lung slices, which was marked in IL-6^−/−^ slices and wild-type (WT) slices treated with IL-6-neutralizing antibody compared with the case of WT slices treated with isotype control [23]. This finding tempts us to consider that IL-6 may also play an antifibrotic role in epithelial injury-based fibrosis. Hence, temporal and spatial differences in IL-6-acting site might affect the pathophysiological outcome in the BLM-injured lung. Indeed, the concept that the role of IL-6 signaling may differ between acute and chronic stages of lung disease is suggested [21]. However, whether IL-6 modulates epithelial injury-based fibrosis in the BLM-instilled mouse model has still not been clarified.

At the early inflammatory stage of BLM-induced lung injury, immunofluorescence studies revealed the predominantly IL-6-acting site and IL-6-producing cell. The phosphorylated forms of Akt and Stat3 were mostly restricted to type 2 pneumocytes, indicating that type 2 pneumocytes respond as the predominantly IL-6-acting site. The phosphorylated form of Stat3 was detected in SP-C^+^ cells even in the PBS-treated lung, which is similar to the result of phospho-protein array showing a clear signal of phospho-Stat3 in the homogenate of PBS-treated lung, suggesting that intrinsic activity of Stat3 may be relatively high in type 2 pneumocytes. On the other hand, type 2 pneumocytes also function as the predominant IL-6-producing cells when activation of Stat3 and Akt was detected in type 2 pneumocytes. Although macrophages could also produce IL-6, their contribution was less than that of type 2 pneumocytes. This finding is supported by a previous report that IL-6 secretion from type 2 pneumocytes is markedly higher than that from alveolar macrophages [39]. These results suggest the possibility that IL-6 induced by BLM affects the cell fate and function of type 2 pneumocytes in an autocrine/paracrine manner, at least in the early inflammatory phase. Then, we examined this possibility using cultured cells.

In both A549 cells and primary cultured type 2 pneumocytes, application of IL-6-neutralizing antibody, a PI3K inhibitor or a Stat3 inhibitor augmented BLM-induced production of cleaved caspases. These results clearly suggest that BLM mobilizes an apoptotic signal in type 2 pneumocytes and simultaneously induces IL-6 production as a compensatory mechanism. Likewise, the IL-6/PI3K/Akt and IL-6/ Stat3 signaling axes can protect alveolar epithelial cells from BLM-induced cell death. This notion is supported by previous reports as follows: blockade of the PI3K/Akt pathway potentiates apoptosis induced by a cyclin-dependent kinase inhibitor in A549 cells [40]; keratinocyte growth factor can inhibit Fas-mediated apoptosis of A549 cells through activation of the PI3K/Akt pathway [41]; Stat3 in type 2 pneumocytes possibly contributes to alveolar epithelial cell survival and surfactant/lipid synthesis, which are necessary for protection of the lung during injury [42]; and the IL-6/Stat3/Akt signaling axis plays a protective role in type 2 pneumocytes [24]. Hence, Akt activated by IL-6 may play a cytoprotective role in concert with Stat3 in type 2 pneumocytes of the lung instilled with BLM. We have confirmed that activation of Akt and Stat3 is induced by BLM in alveolar type II cells within a few hours, which is relatively faster than the time course of BLM-induced IL-6 synthesis/release (data not shown). This finding indicates that IL-6-independent and BLM-induced PI3K/Akt and Stat3 activation also exist in alveolar type II cells and can explain the finding that the apoptotic signal augmented by a PI3K inhibitor or a Stat3 inhibitor was stronger than that augmented by IL-6-neutralizing antibody. However, the effect of IL-6-neutralizing antibody suggests that at least IL-6-dependent Akt and Stat3 activation can function as a survival signal in BLM-treated type 2 pneumocytes in an autocrine/paracrine manner. Moreover, application of IL-6-neutralizing antibody to type 2 pneumocytes also affected their cytokine-producing activity under stimulation with BLM. The BLM-induced production of several cytokines such as GM-CSF, IL-2, IL-9, IL-13, MCP-1 and THPO was modulated by endogenous IL-6 in type 2 pneumocytes. GM-CSF and IL-9 play a protective role against BLM-induced lung fibrosis through a prostaglandin-dependent mechanism [43, 44]. In contrast, IL-13 and MCP-1 contribute to the development of BLM-induced lung fibrosis [45, 46]. On the other hand, IL-2 is shown to be involved not in fibrosis but in lymphocytic infiltration in the lung instilled with BLM [47]. THPO has not been shown to be related to lung injury, but plays a protective role in liver fibrosis [48]. Hence, the stimulatory effect of IL-6 on expression of antifibrotic cytokines, IL-9 and THPO, and the inhibitory effect of IL-6 on expression of proinflammatory and profibrotic cytokines, IL-2 and IL-13, may improve the microenvironment in the lung exposed to BLM. In conjunction with the cytoprotective activity of IL-6, at least IL-6 was upregulated predominantly in type 2 pneumocytes at the early inflammatory stage, and may exert counter effects on the development of BLM-induced lung injury. In the BLM-instilled lung, IL-6 was induced at both the early inflammatory stage and early fibrotic stage. Then, we further investigated how blockade of IL-6 at each stage affects the pathophysiological outcome of BLM-induced lung injury.

According to our expectation, blockade of IL-6 at the early inflammatory stage of BLM-induced lung injury enhanced apoptosis of type 2 pneumocytes. In addition, an increase in population of neutrophils in BALF from BLM-instilled mice was also enhanced by IL-6-neutralizing antibody but not isotype control. Although a precise evaluation of the inflammatory cell populations in the lung parenchyma contributing to the severity of fibrosis is needed [20], the increase in neutrophils in BALF suggests that blockade of IL-6 at the early inflammatory stage of BLM-induced lung injury at least enhances lung inflammation. Furthremore, even at the transitional period from the inflammatory phase to the fibrotic phase, the application of IL-6-neutralizing antibody to the lung instilled with BLM resulted in obvious fibrosis. The exact reason why blockade of IL-6 at the early inflammatory stage of BLM-induced lung injury enhanced fibrotic formation at 7 dpi but not 14 dpi is unclear in the present study. At 14 dpi, alveoli nearly obliterated with fibrous masses that are Grade 7 by Ashcroft score were observed more frequently in BLM + anti-IL-6 group than both BLM and BLM + control IgG groups. Hence, histopathological observation or biochemical analysis more than 14 days may be needed. In the normal alveoli of most patients with IPF, numerous type 2 pneumocytes actively undergo programmed cell death, suggesting epithelial injury-based mechanisms of lung fibrosis [49]. This concept has been clearly validated by the finding that induction of type 2 pneumocyte-specific cell death by a genetically engineered method leads to pulmonary fibrosis [15]. Taking these findings together, IL-6 at the early inflammatory stage of BLM-induced lung injury functions as an inhibitory factor in the epithelial injury-based mechanisms of lung fibrosis. The question arose as to whether blockade of the protective role of IL-6 could affect BLM-induced TGF-β1 expression because a correlation between IL-6 signal and TGF-β1 expression has been shown in BLM-challenged mice [19]. It is well known that TGF-β/smad3 signaling can stimulate fibroblast differentiation and epithelial mesenchymal transition and that mouse with Smad3-deficiency shows resistance to BLM-induced lung fibrosis [50]. Moreover, a genetic inhibition of TGF-β/TβRII signaling axis in alveolar type II cells limits BLM-induced fibrogenesis by increasing fibroblast apoptosis [51]. Thus, TGF-β1 is closely associated with epithelial cell fate and subsequent fibrotic formation. Blockade of IL-6 at the early inflammatory stage of BLM-induced lung injury significantly enhanced BLM-induced TGF-β1 mRNA expression at 7 dpi (Additional file 2: Figure S2). Although the precise mechanism of this finding is still unclear, the upregulation of BLM-induced TGF-β1 may partly contribute to obvious fibrotic formation manifested by blocking IL-6 at the early inflammatory stage of BLM-induced lung injury.

In contrast to the case of blocking the first peak of IL-6, blockade of IL-6 at the early fibrotic stage of BLM-induced lung injury improved both the body weight loss and the survival rate of mice. Likewise, BLM-induced lung fibrosis was significantly inhibited by IL-6-neutralizing antibody at 14 dpi. The possibility that blockade of IL-6 at the early fibrotic stage of BLM-induced lung injury simply delayed the onset of BLM-induced fibrotic formation remains. In a preliminary study, however, severe fibrosis induced by BLM was not observed up to 40 dpi in mice administered with IL-6-neutralizing antibody. These results suggest that IL-6 positively contributes to lung fibrosis under a fibrosis-establishing state. This finding showed good agreement with previous reports that lung fibrosis is ameliorated by genetic or pharmacologic blockade of IL-6 [19, 21]. In addition, the abundant localization of phospho-Stat3 in fibrotic areas at the early fibrotic stage of BLM-induced lung injury was in accord with previous reports on lung sections from both mice with lung fibrosis and patients with IPF [20, 22]. It has been clearly shown that IL-6/gp130/Stat3 signaling axis in lung fibroblasts derived from IPF patients enhances the resistance to apoptosis by upregulating Bcl-2 expression [52]. Likewise, viral delivery of oncostatin M, one of IL-6 family members sharing gp130-signaling subunit, to the lung induced severe fibrosis associated with Stat3 activation in a TGF-β/smad3-independent manner [20, 53]. Hence, localization of activated Stat3 in fibrotic area may be one of pathophysiological indices in IL-6 family-mediated fibrogenesis. At the early fibrotic stage of BLM-induced lung injury, the IL-6-producing cells were mainly macrophages and fibroblasts but not type 2 pneumocytes, and phospho-Stat3 was not observed in type 2 pneumocyte. Thus, an autocrine/paracrine loop of IL-6 signaling was not observed in type 2 pneumocytes. The changes in IL-6-producing and -acting cells between different injury stages may at least support a bidirectional role of IL-6 in BLM-induced lung fibrosis.

Targeting IL-6 is a rational approach to various autoimmune and chronic inflammatory diseases [54]. Likewise, the IL-6/gp130/Stat3 signaling axis is expected to be a new therapeutic target in IPF [20, 22]. Furthermore, neutralization of IL-6, especially at the fibrotic stage of lung injury, significantly inhibits the progression of lung fibrosis [21]. Thus, an anti-IL-6 strategy may be beneficial for IPF patients. However, recent clinical case reports presented patients with established rheumatoid arthritis (RA) with RA-associated interstitial lung disease (ILD) treated with tocilizumab, an anti-IL-6 receptor monoclonal antibody, who had an acute exacerbation of interstitial infiltrates or pulmonary fibrosis [55, 56]. Based on the accumulating information on clinical cases, the effects of an anti-IL-6 strategy on ILD should be observed carefully.

## Conclusion

In the present study, we clearly demonstrated that the role of IL-6 signaling could differ between the inflammatory and fibrotic stages of BLM-induced lung injury. In particular, blockade of IL-6 at the early inflammatory stage of BLM-induced lung injury can lead to apoptosis and functional change of type 2 pneumocytes and accelerate lung fibrotic formation partly by regulating TGF-β1 expression. BLM eventually effectively induces lung fibrosis, suggesting that the protective role of IL-6 as a compensatory mechanism cannot intrinsically overcome BLM-induced lung fibrosis and is masked in the final pathological outcome. Considering an anti-IL-6 strategy against lung inflammatory disease, however, this compensatory mechanism could be a crucial element in management of the disease.

## Additional files

Additional file 1: Figure S1. Blockade of IL-6 at early inflammatory stage of BLM-injured lung affects SMA induction in the lung. Mice were intratracheally administered with IL-6-neutralizing antibody or isotype control at 6, 30 and 54 h (0, 1 and 2 dpi) after instillation of BLM. Then, the left lung lobes were dissected out at 5, 7 and 14 dpi. The left lung lobes of untreated mice were used as negative control (N.C.). The lobe was longitudinally cut into two pieces, each of which was used for protein preparation and total RNA preparation, respectively. Total RNA was used for determination of TGF-β1 mRNA (See Figure S2A). Protein samples (5 μg) were subjected to Western blot analysis with anti-SMA and anti-β-actin. The signal of each sample was determined by using a densitometer and normalized to each internal control (β-actin). Signal values were expressed as relative fold induction to the N.C. #1 (1.0). Similar results were obtained in two independent experiments. (PDF 1495 kb) Additional file 2: Figure S2. Blockade of IL-6 at early inflammatory stage of BLM-injured lung affects TGF-β1 induction in the lung. *A)* Total RNA from each sample corresponding to the experimental condition in Figure S1 was subjected to RT-PCR for amplification of TGF-β1 and GAPDH cDNAs. The specific primers and the settings of the thermal cycler employed here were accordant with our previous report [Ref. 5, Yamauchi K, et al.]. The amplified products were separated on a 1.5 % agarose gel and visualized with ethidium bromide staining under UV radiation. The signal of each sample was determined by using a densitometer and normalized to each internal control (GAPDH). Signal values were expressed as relative fold induction to the N.C. #1 (1.0). Similar results were obtained in two independent experiments. At 7 dpi, blockade of IL-6 positively regulated the BLM-induced TGF-β1 mRNA expression. Then, we further elucidated this possibility. *B)* The whole lung lobes from three groups of mice (PBS group, BLM+control IgG group and BLM+anti-IL-6 group) were dissected out at 7 dpi. Total RNA (0.1 μg) from each sample was subjected to RT-PCR for amplification of TGF-β1 and GAPDH cDNAs, and each densitometric signal was obtained. Normalized signal values were expressed as relative fold induction to one of the values in PBS group. Data are shown as mean ± S.E.M. (*n*=6). **P* < 0.05, significantly different between values of three groups (ANOVA followed by Tukey's test). (PDF 1290 kb)
