# Supplementary material for: Bidirectional role of IL-6 signal in pathogenesis of lung fibrosis
Source: Respir Res. 2015 Aug 20;16(1):99. doi: 10.1186/s12931-015-0261-z (PMC4546032; doi:10.1186/s12931-015-0261-z)
Supplement: Additional file 1: Figure S1. — Blockade of IL-6 at early inflammatory stage of BLM-injured lung affects SMA induction in the lung. Mice were intratracheally administered with IL-6-neutralizing antibody or isotype control at 6, 30 and 54 h (0, 1 and 2 dpi) after instillation of BLM. Then, the left lung lobes were dissected out at 5, 7 and 14 dpi. The left lung lobes of untreated mice were used as negative control (N.C.). The lobe was longitudinally cut into two pieces, each of which was used for protein preparation and total RNA preparation, respectively. Total RNA was used for determination of TGF-β1 mRNA (See Figure S2A). Protein samples (5 μg) were subjected to Western blot analysis with anti-SMA and anti-β-actin. The signal of each sample was determined by using a densitometer and normalized to each internal control (β-actin). Signal values were expressed as relative fold induction to the N.C. #1 (1.0). Similar results were obtained in two independent experiments. (PDF 1495 kb) [file 12931_2015_261_MOESM1_ESM.pdf]

Additional File 1 (Kobayashi T, et al.)

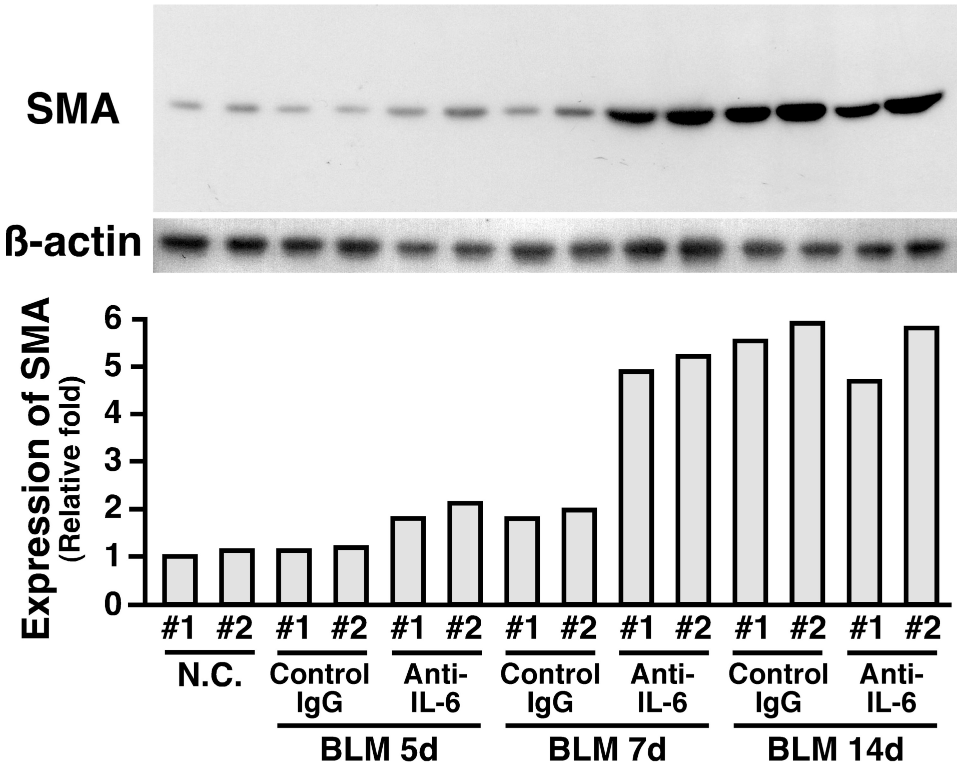

Figure S1. Blockade of IL-6 at early inflammatory stage of BLM-injured lung affects SMA induction in the lung.

Mice were intratracheally administered with IL-6-neutralizing antibody or isotype control at 6, 30 and 54 h (0, 1 and 2 dpi) after instillation of BLM. Then, the left lung lobes were dissected out at 5, 7 and 14 dpi. The left lung lobes of untreated mice were used as negative control (N.C.). The lobe was longitudinally cut into two pieces, each of which was used for protein preparation and total RNA preparation, respectively. Total RNA was used for determination of TGF-β1 mRNA (See Figure S2A). Protein samples (5 µg) were subjected to Western blot analysis with anti-SMA and anti-β-actin. The signal of each sample was determined by using a densitometer and normalized to each internal control (β-actin). Signal values were expressed as relative fold induction to the N.C. #1 (1.0). Similar results were obtained in two independent experiments.
