# Supplementary material for: Bidirectional role of IL-6 signal in pathogenesis of lung fibrosis
Source: Respir Res. 2015 Aug 20;16(1):99. doi: 10.1186/s12931-015-0261-z (PMC4546032; doi:10.1186/s12931-015-0261-z)
Supplement: Additional file 2: Figure S2. — Blockade of IL-6 at early inflammatory stage of BLM-injured lung affects TGF-β1 induction in the lung. A) Total RNA from each sample corresponding to the experimental condition in Figure S1 was subjected to RT-PCR for amplification of TGF-β1 and GAPDH cDNAs. The specific primers and the settings of the thermal cycler employed here were accordant with our previous report [Ref. 5, Yamauchi K, et al.]. The amplified products were separated on a 1.5 % agarose gel and visualized with ethidium bromide staining under UV radiation. The signal of each sample was determined by using a densitometer and normalized to each internal control (GAPDH). Signal values were expressed as relative fold induction to the N.C. #1 (1.0). Similar results were obtained in two independent experiments. At 7 dpi, blockade of IL-6 positively regulated the BLM-induced TGF-β1 mRNA expression. Then, we further elucidated this possibility. B) The whole lung lobes from three groups of mice (PBS group, BLM+control IgG group and BLM+anti-IL-6 group) were dissected out at 7 dpi. Total RNA (0.1 μg) from each sample was subjected to RT-PCR for amplification of TGF-β1 and GAPDH cDNAs, and each densitometric signal was obtained. Normalized signal values were expressed as relative fold induction to one of the values in PBS group. Data are shown as mean ± S.E.M. (n=6). *P < 0.05, significantly different between values of three groups (ANOVA followed by Tukey's test). (PDF 1290 kb) [file 12931_2015_261_MOESM2_ESM.pdf]

Additional File 2 (Kobayashi T, et al.)

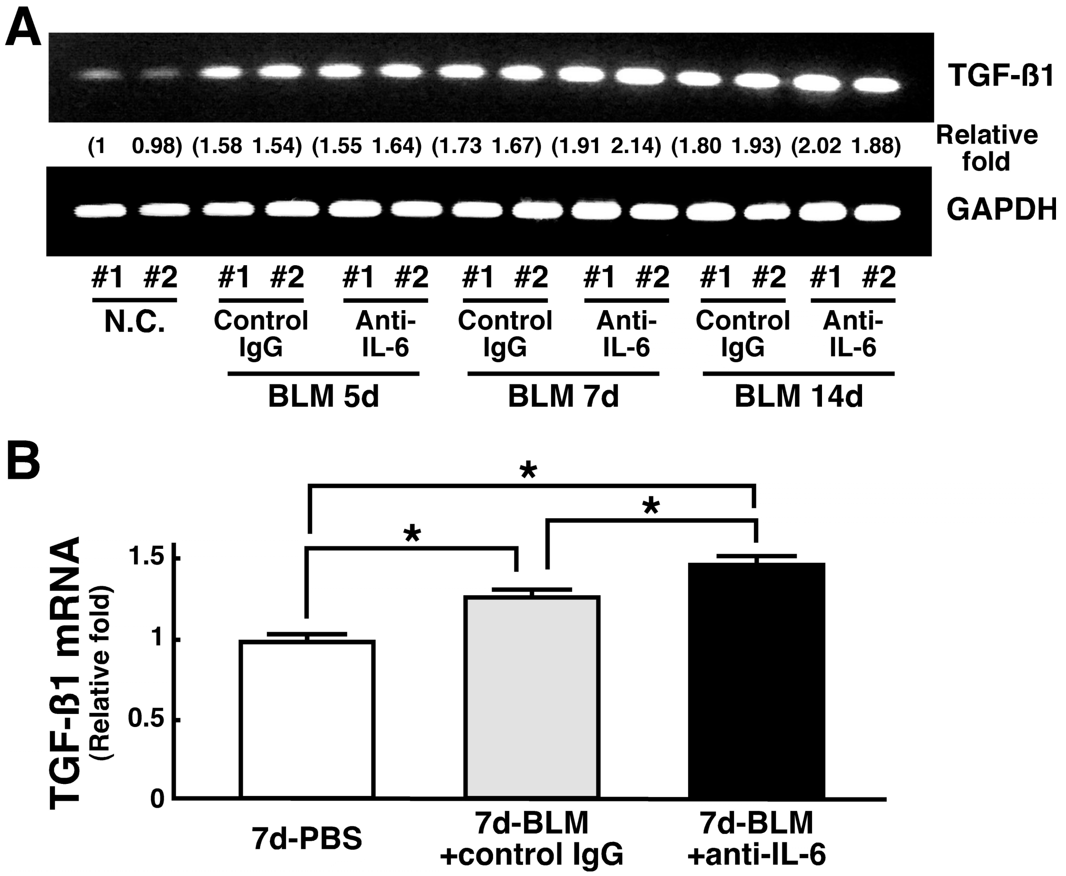

Figure S2. Blockade of IL-6 at early inflammatory stage of BLM-injured lung affects TGF-β1 induction in the lung.

A) Total RNA from each sample corresponding to the experimental condition in Figure S1 was subjected to RT-PCR for amplification of TGF-β1 and GAPDH cDNAs. The specific primers and the settings of the thermal cycler employed here were accordant with our previous report [Ref. 5, Yamauchi K, et al.]. The amplified products were separated on a 1.5% agarose gel and visualized with ethidium bromide staining under UV radiation. The signal of each sample was determined by using a densitometer and normalized to each internal control (GAPDH). Signal values were expressed as relative fold induction to the N.C. #1 (1.0). Similar results were obtained in two independent experiments. At 7 dpi, blockade of IL-6 positively regulated the BLM-induced TGF-β1 mRNA expression. Then, we further elucidated this possibility. ***B)*** The whole lung lobes from three groups of mice (PBS group, BLM+control IgG group and BLM+anti-IL-6 group) were dissected out at 7 dpi. Total RNA (0.1 μg) from each sample was subjected to RT-PCR for amplification of TGF-β1 and GAPDH cDNAs, and each densitometric signal was obtained. Normalized signal values were expressed as relative fold induction to one of the values in PBS group. Data are shown as mean ± S.E.M. (n=6). **P* < 0.05, significantly different between values of three groups (ANOVA followed by Tukey test).
